# A phase I/II radiation dose escalation trial using simultaneous integrated boost technique with elective nodal irradiation and concurrent chemotherapy for unresectable esophageal Cancer

**DOI:** 10.1186/s13014-019-1249-5

**Published:** 2019-03-15

**Authors:** Chen Li, Wenjie Ni, Xin Wang, Zongmei Zhou, Wei Deng, Xiao Chang, Dongfu Chen, Qinfu Feng, Jun Liang, Xiaozhen Wang, Lei Deng, Wenqing Wang, Nan Bi, Tao Zhang, Zefen Xiao

**Affiliations:** Department of Radiation Oncology, National Cancer Center/ National Clinical Research Center for Cancer/ Cancer Hospital, Chinese Academy of Medical Sciences and Peking Union Medical College, No.17 Panjiayuan Nanli, Beijing, 100021 China

**Keywords:** Esophageal squamous cell carcinoma, Concurrent Chemoradiotherapy, Dose-escalation, Simultaneous integrated boost, Elective nodal irradiation

## Abstract

**Background:**

To investigate the safety and tolerability of simultaneous integrated boost (SIB) technique concurrent with elective nodal irradiation (ENI) and dual-drug chemotherapy for patients with unresectable esophageal cancer.

**Methods:**

In phase I, the prophylactic PTV received a stable dose of 50.40Gy/1.80Gy/28f while the boost area was planned with 3 consecutive dose levels: the first dose level was 60.76Gy/2.17Gy/28f, and then escalated approximately every 2 Gy. ENI was incorporated in Clinical Target Volume (CTV), and paclitaxel and nedaplatin were given concurrently for at least 5 weeks. In phase II, enrolled patients were treated with Maximum Tolerated Dose (MTD) obtained in phase I and the compliance rate, survival results and toxicities were evaluated.

**Results:**

From December 2014 to April 2017, 53 patients were enrolled. In phase I, 2 out of 6 patients developed Dose-Limiting Toxicity (DLT) at dose level 1. Due to excessive treatment-related toxicities, the escalation process was suspended and de-escalated to 59.92Gy /2.14Gy /28 f. Three patients were treated at this dose level, all of whom completed at least 5 weeks of chemotherapy and none of whom reached a DLT, determining the newly added dose level to be the MTD.

In phase II, 44 patients were treated with MTD, 31 of them (70.0%) completed at least 5 weeks of chemotherapy. The most common Grade 3 or 4 toxicities in phase II included leukopenia (21%) and esophagitis (15%). With a median follow-up time of 16.9 months, 1-y OS, DFS and local failure-free survival were 76.9, 63.6 and 78.8% respectively.

**Conclusion:**

The SIB technique was feasible and safe at the MTD (95% PGTV/PTV 59.92/50.40Gy/28f) concurrent with ENI and dual-drug chemotherapy for patients with unresectable esophageal cancer.

**Trial registration:**

clinicaltrials.gov NCT02429622. Retrospectively registered on April 24, 2015.

**Electronic supplementary material:**

The online version of this article (10.1186/s13014-019-1249-5) contains supplementary material, which is available to authorized users.

## Background

Esophageal cancer (EC) is the sixth leading cause of cancer-related death worldwide. Since RTOG 85–01 [1, 2], concurrent chemoradiotherapy (CCRT) has become one of the standard treatment regimens for patients with locally advanced EC. Currently, many research institutions adopt the dose of 50.4Gy based on RTOG94–05 [3], which shows that patients in the high-dose group (64.8Gy) have no improvement in terms of overall survival (OS) or local control compared with patients in the low-dose group (50.4Gy).

However, the dose proposed by RTOG94–05 remains controversial nowadays because of several limitations. Firstly, RTOG94–05 was conducted in the age of two-dimensional radiotherapy (2DRT), since when a number of changes in treatment have occurred, including the application of three-dimensional radiotherapy (3DRT) and intensity modulated radiotherapy (IMRT). Moreover, some retrospective studies [4, 5] have demonstrated that local control was unsatisfying in patients treated with 50.4Gy and suggested higher radiation doses.

To achieve high dose of irradiation of on tumor without significantly increasing the exposure of organs at risk (OAR), the SIB technique is generally employed, which can give a conformable heterogeneous dose distribution inside one radiation field simultaneously. This technique has been successfully implemented to treat cancers of various regions such as head and neck, prostate, cervix, etc. [6–9]. Many studies on SIB has been done for EC in terms of dosimetry [10–12], proving that it can increase the dose in high-risk areas without increasing the irradiation of normal tissues. Moreover, SIB can significantly reduce the workload for both physicians and physicists in terms of simulation, delineation and planning when compared to “shrinking-field technique” [7].

However, when SIB is applied, high-risk areas will receive a single dose of more than 2Gy/f. For cavity organ like esophagus, such high fraction size may cause serious consequences such as esophageal perforation, severe radiation-related esophagitis, etc. Therefore, before large-scale application of this technique, evidences from Phase I to III clinical trial are still needed. Some relevant phase I-II studies were reported in the literature [13–16]. Specifically, Yu. et al. [13] published the first report of the clinical application of SIB technique and set 63Gy/2.25Gy/28f as the dose for their boost targets. Fu. et al. [14] then introduced PET/CT simulation into the treatment of EC and escalated high FDG uptake regions to 70Gy. Chen, et al. adopted the dose mode of 66Gy/2.2Gy/30f to gross tumor and 54Gy/1.8Gy/30f to subclinical diseases [15]. In a most recently published article from Welsh JW, et al. [16], the MTD was evaluated to 63Gy to the boost area. However, one thing that should not be omitted is that the majority of patients in the studies aforementioned use the Involved Field Radiotherapy (IFRT), and studies in the context of Elective Node Irradiation (ENI) are still missing to the best of our knowledge. The MTD determined under IFRT cannot be applied directly to the case of ENI before further justification because ENI uses a much larger radiation field. Therefore, the main purpose of this research is to explore the MTD and feasibility of SIB technique in the context of ENI and concurrent chemotherapy (paclitaxel and nedaplatin).

## Materials and methods

### Eligibility criteria

Patients (18 ≤ age<70) with histologically or cytologically confirmed primary squamous cell carcinoma (SCC) of the esophagus were eligible for the study. All patients were required to be unsuitable for surgery according to the assessment prior to or during chemoradiation by thoracic surgeons or a multi-disciplinary team (MDT). All patients enrolled were required to be candidates for potential curative treatment, with a Karnofsky performance score (KPS) ≥ 70 and had no distant visceral metastasis or prior treatment history. Patients with a history of a prior malignancy were considered eligible if disease-free for 5 or more years. Patients enrolled must have adequate bone marrow, liver and kidney function. The protocol was reviewed and approved by the Institutional Review Board. Written informed consent was obtained from each patient.

### Pretreatment evaluation

Pretreatment evaluation included history-taking, physical examination, electrocardiogram and assessment of bone marrow, renal, hepatic and pulmonary functions. Local and systematic evaluation of the tumor included barium esophagram, computed tomography (CT) of neck, chest and abdomen, and upper endoscopy with endoscopic ultrasonography (EUS) and biopsy. PET/CT was recommended but not mandatory for patients enrolled. Bone scan was performed in case of bone pain or abnormally elevated serum alkaline phosphatase (ALP), and cranial MRI was performed if clinically indicated. Patients were required to receive one week of intravenous antibiotics and nutrition support as prevention if one of the following characteristics present: a. Deep ulceration or other signs indicating possibility of future perforation shown on esophagram; b. Heavy tumor burden such as T4 disease; c. Heterogeneous tumor signal or other signs indicating necrosis inside tumor on CT images. Because of the poor coincidence rate in terms of lymph node metastasis between postoperative pathological reports and preoperative assessment [17], the 6th edition tumor-node-metastasis (TNM) staging system from American Joint Committee on Cancer (AJCC) was adopted for its exclusion of the exact number of metastatic lymph nodes in its system.

### Radiation therapy and chemotherapy

**Fig. 1:**
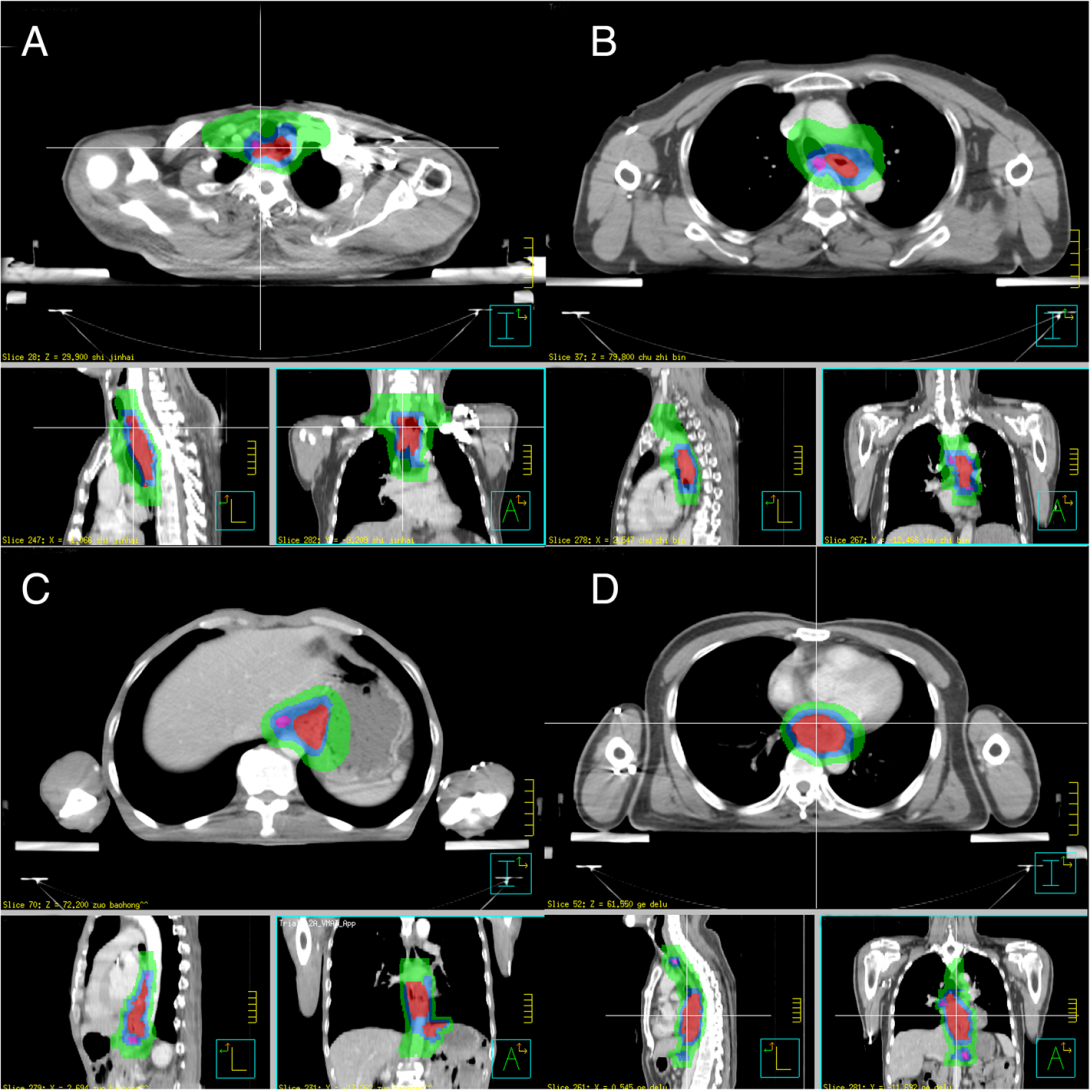
Targets contouring of **a** Upper thoracic esophagus (Ut); **b** Middle thoracic esophagus (Mt); **c** Lower thoracic esophagus (Lt); **d** Middle thoracic esophagus (Mt) with supraclavicular and abdominal lymph node metastasis. Each contour is displayed on the axial, coronal, and sagittal planes through the primary tumor. Red shading indicates GTV-T, the pink line area includes GTV-N, the green line outlines PTV, the blue line outlines PGTV

Treatment-planning CT scans using intravenous contrast were performed for all patients in the supine position with both arms straight beside the body. Gross Tumor Volume (GTV-T), defined as any visible primary tumor, was delineated by physicians using all possible resources (CT, EUS, barium esophagram and 18FDG PET/CT, etc.). The metastatic regional nodes (GTV-N) was defined as any lymph node diagnosed as or highly-suspected as metastatic. PGTV was created by expanding GTV-T and GTV-N by 1 .0cm craniocaudally and by 0 .5cm laterally. ENI was adopted for all patients, meaning that all patients had their prophylactic lymph node regions irradiated and no patients had interruption within their Clinical Target Volume (CTV). The typical contouring of CTVs for tumor in different locations are depicted and described in Fig. 1 and Table 1 respectively (refer to International Association for the Study of Lung Cancer (IASLC) Lymph Node Map [18] for exact definition of each lymph node region). PTV was generated using a uniform 0.5 cm expansion around CTV.

**Table 1:** The delineation of CTV for tumor in different location

Location	Upper margin	Lower margin	Lymph node stations included in CTV
Upper thoracic esophagus (Ut)	Cricothyroid membrane / 1–1 .5cm superior to the highest metastatic lymph node	3 cm inferior to the lower margin of the tumor / 1–1 .5cm inferior to the lowest metastatic lymph node	supraclavicular, station 1R, 1 L, 2R, 2 L, 4R, 4 L, 7 (station 3, 8, paracardial and left gastric lymph nodes may be included if metastatic lymph nodes exist)
Middle thoracic esophagus (Mt)	First thoracic vertebra / 1–1 .5cm superior to the highest metastatic lymph node	3 cm inferior to the lower margin of the tumor / 1–1 .5cm inferior to the lowest metastatic lymph node	supraclavicular, station 1R, 1 L, 2R, 2 L, 4R, 4 L, 7, 8 (station 3, paracardial and left gastric lymph nodes may be included if metastatic lymph nodes exist)
Lower thoracic esophagus (Lt)	3-5 cm superior to the tumor / 1–1 .5cm superior to the highest metastatic lymph node	3 cm inferior to the lower margin of the tumor / 1–1 .5cm inferior to the lowest metastatic lymph node	station 2R, 2 L, 4R, 4 L, 7, 8, paracardial and left gastric lymph nodes (supraclavicular, staion1R, 1 L, 3 may be included if metastatic lymph nodes exist)

**Fig. 2:**
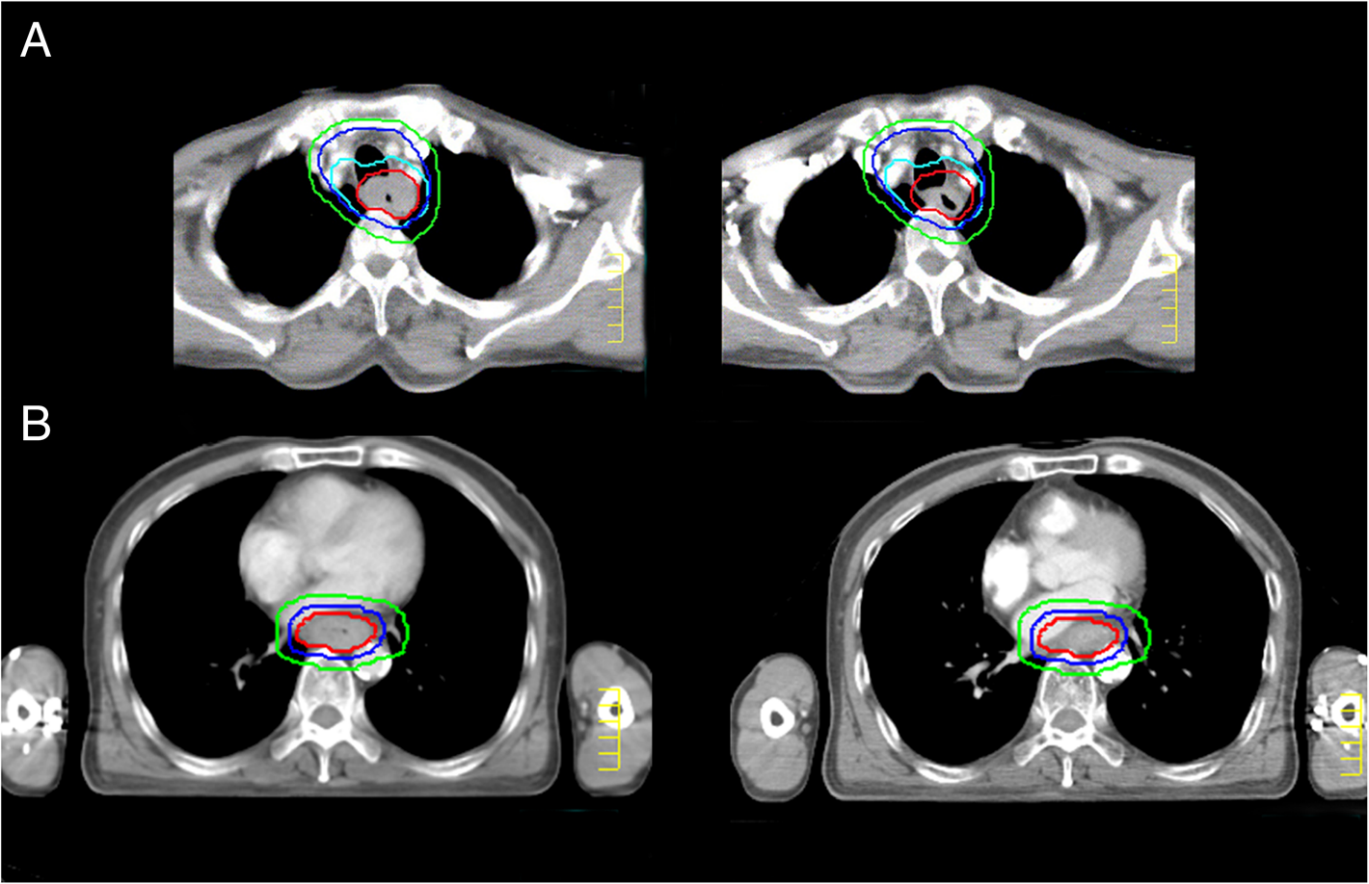
CT-CT fusion images of two enrolled patients. Images on the left are pre-treatment ones, while those on the right are taken after 23 fractions of radiation. Red line indicates GTV-T, the green line outlines PTV, the blue line outlines PGTV

Radiotherapy plans were generated by the Pinnacle treatment planning system (version 8.0 m). Irradiation was delivered with 6-MV photon energy using a linear accelerator. Dose coverage required that 95% of PTVs receive the prescribed dose. Dose constraints to OARs were detailed in Additional file 1: Table S1. To ensure that both primary tumor and metastatic lymph nodes always stay within the boost region (PGTV), all patients were required to be re-scanned after 21–23 fractions and two sets of CT images would be fused together (shown in Fig. 2) to evaluate the shrinkage and movement of all targets. The radiotherapy plan would be adjusted accordingly to ensure the full-dose irradiation on the primary tumor.

All patients received weekly infusion of paclitaxel (50 mg/m^2^) and nedaplatin (25 mg/m^2^) concurrent with radiotherapy for at least 5 weeks.

### Toxicity evaluation

Toxicities were scored according to National Cancer Institute Common Terminology Criteria for Adverse Events (CTCAE) and evaluated weekly during treatment. DLT was defined as grade 4 or higher hematological toxicities (including anemia, neutropenia and thrombocytopenia) and/or grade 3 or higher non-hematological toxicities (including radiation-induced esophagitis, pneumonitis, gastrointestinal reaction, dermatitis, weight loss, hepatic and renal dysfunction, etc.). In case of grade 3 hematologic toxicity, radiotherapy would continue with the dose of chemotherapy reduced by 25% since the next cycle. In case of DLT, both radiotherapy and chemotherapy were discontinued at the same time until patient recovered to grade 0–1 toxicity and the chemotherapeutic drugs would not be administered thereafter.

### Radiation dose escalation

Prescribed dose was assigned according to the pre-designed dose-escalation scheme. The prophylactic PTVs received a stable dose of 50.40Gy /1.8Gy /28f, while the therapeutic PGTVs were planned to be prescribed with three consecutive dose levels: 60.76Gy /2.17Gy /28f (dose level 1), 61.88Gy /2.21Gy /28f (dose level 2) and 64.12Gy /2.29Gy /28f (dose level 3). Radiation dose escalation was carried out according to following rules. At each dose level, a minimum of three patients were treated and observed for at least three months after completion of treatment. If DLT was not present, the escalation process wound continue to next dose level. In case DLT was observed in two or more patients, the dose escalation would be terminated and MTD was determined as the former dose level. If only one patient developed DLT, then another three patients would be accrued at the same dose level. In case of further DLT present, the escalation process would be terminated and the previous dose level was then determined as MTD.

### Statistical analysis

The primary endpoint of Phase I was to establish MTD. The primary endpoint of Phase II part was to evaluate the completion rate and safety of this MTD. Secondary endpoints of Phase II part included treatment-related toxicities, OS, Disease Free Survival (DFS) and local failure-free survival, etc. Continuous variables were summarized by descriptive statistics such as means, standard deviations, medians, and ranges. Categorical variables were tabulated by frequency and percentage. Survival functions were calculated from the date of treatment using Kaplan–Meier estimates. Statistical calculations were performed with the Statistical Package for Social Sciences software 23.0 (SPSS Inc., Chicago, IL).

### Follow-up and evaluation

The first follow-up was performed one month after completion of treatment, after which the interval was set to be every three months for the first two years and every six months thereafter. Physical examination, barium esophagram, CT scan of neck, chest and abdomen, and ultrasonography of neck and supraclavicular lymph nodes regions were performed at each visit. If esophageal recurrence was suspected according to barium esophagram or CT, endoscopy and biopsy or PET/CT were used for confirmation.

## Results

### Patient Characteristics & Dose–volume parameters

A total of 53 patients with SCC were enrolled in this study**.** Characteristics of these patients and dose-volume parameters are shown in Table 2 in detail. Most patients (89%) were male and had stage IV (44%) or III (43%) diseases. Among those stage IV patients, 4 (17%) patients had metastatic lymph nodes in both supraclavicular and abdominal regions, 2 (9%) patient only had metastatic abdominal lymph nodes, and 17 (74%) patients only had metastatic supraclavicular lymph nodes. As for general conditions, among all stage IV patients, KPS of 100, 90, and 80 accounted for 4, 57, and 39%, respectively, similar to that of non- stage IV patients.

**Table 2:** Patient and treatment characteristics

Characteristic	No. of patients	%
Total patients	53	100
Age, years		
Median	58	
Range	41–69	
Gender		
Female	6	11
Male	47	89
Karnofsky Performance Status		
100	1	2
90	29	55
80	22	41
70	1	2
Tumor Length, cm		
Median	6	
Range	2–14	
Length<3 cm	2	4
3 cm ≤ Length<5 cm	12	23
5 cm ≤ Length<10 cm	35	66
Length ≥ 10 cm	4	7
Clinical Stage (UICC 2002)		
T stage		
T1	3	6
T2	7	13
T3	21	40
T4	22	41
N stage		
N0	1	2
N1	52	98
TNM stage		
IIA	1	2
IIB	6	11
III	23	43
IVA	10	19
IVB	13	25
Radiotherapy Technique		
SIB-IMRT	35	66
SIB-VMAT	18	34
GTV volume (cm^3^)		
Median	41.77	
Range	6.30–144.04	
GTV-nd volume (cm^3^)	10.53	
	0.00–46.67	
PGTV volume (cm^3^)		
Median	175.54	
Range	36.59–374.09	
CTV volume (cm^3^)		
Median	364.72	
Range	172.47–562.21	
PTV volume (cm^3^)		
Median	630.91	
Range	325.14–854.99	

### Maximum Tolerated Dose & Treatment compliance

At dose level 1 (95% PTV/PGTV 60.76/50.4Gy), one of the first three patients developed grade 4 thrombocytopenia as well as esophageal perforation, then another group of three patients were recruited. However, one patient from the second group developed grade 3 esophagitis. Thus, we suspended the escalation process and lowered the dose level of PGTV to 59.92Gy/2.14Gy/28 f. Three patients were treated in the lowered dose level and none of them reached a DLT. Considering the DLTs developed in previous level 1, we determined the newly added lowered dose level to be the MTD.

Phase II trial started at April, 2015 and ended at April, 2017. Forty four patients were enrolled during this period, all of whom accepted SIB with the recommended dose (95% PTV/PGTV 59.92/50.40Gy) and concurrent chemotherapy of paclitaxel and nedaplatin. Six patients did not complete both chemotherapy and radiotherapy, accounting for 14% of all 44 patients. 9 (20%) patients did not finish radiotherapy while 13 (30%) patients did not finish chemotherapy.

The causes of non-compliance to either chemotherapy or radiation therapy were due to severe esophagitis or upper gastrointestinal reaction in 4 patients, esophageal perforation in 1 patient and comorbidities (chronic pulmonary disease) in 1 patient**.** As for those who only finished chemotherapy**,** 4 patients were due to severe esophagitis or upper gastrointestinal reaction, 1 to esophageal perforation, 1 to pneumonitis, 1 to patient refusal and 2 to comorbidities (chronic pulmonary disease). Among those who only finished radiation therapy, 8 patients were due to myelosuppression, 4 patients were due to severe esophagitis or upper gastrointestinal reaction, 1 to esophageal perforation. Among the patients who did not complete radiotherapy, 4 (44%) patients had 25 fractions (PGTV/PTV ≥ 53.5/45Gy) or more, 2 (22%) had 20–25 fractions (42.8/36Gy ≤ PGTV/PTV<53.5/45Gy) while 3 (33%) had less than 20 fractions (PGTV/PTV<42.8/36Gy). Among the patients who did not finish chemotherapy, 7 (54%) patients completed 4 weeks of chemotherapy, 4 (31%) patients received 3 weeks, while only 2 (15%) patients had less than 3 weeks of chemotherapy during radiation.

Besides, according to patients’ CT-CT fusion images at 21–23 fractions, it is found that despite of tumor shrinkage, high-risk areas still stayed within PGTV, which ensuring that they receive full-dose prescription.

### Treatment-related toxicity

Details of treatment-related toxicities are listed in Table 3. All patients were evaluable for acute toxicity. The most common grade1–2 acute toxicities were radiation esophagitis and skin-reaction, both occurring in 84% of all patients. Grade 1–2 leukopenia was also very common, occurring in 66% of all patients. As for the most common grade 3–4 acute toxicities, leukopenia happened in 21% of all patients while esophagitis in 15%. As for late toxicities (≥90 days), three patients developed esophageal perforation and one patient died of esophageal hemorrhage (113 days after treatment).

**Table 3:** Treatment-related toxicity

Toxicity	Grade 1		Grade 2		Grade 3		Grade 4		Grade 5	
	*n*	%	*n*	%	*n*	%	*n*	%	*n*	%
Acute										
Leukopenia	18	(34)	17	(32)	11	(21)	0	(0)	0	(0)
Anemia	11	(21)	0	(0)	1	(2)	0	(0)	0	(0)
Thrombocytopenia	6	(11)	6	(11)	1	(2)	1	(2)	0	(0)
Skin Reaction	33	(62)	12	(23)	0	(0)	0	(0)	0	(0)
Esophagitis	26	(49)	19	(36)	7	(13)	1	(2)	0	(0)
Weight Loss	15	(28)	0	(0)	0	(0)	0	(0)	0	(0)
Upper G.I.	15	(28)	2	(4)	5	(9)	0	(0)	0	(0)
Pneumonitis	1	(2)	0	(0)	3	(6)	0	(0)	0	(0)
Tracheitis	17	(32)	2	(4)	0	(0)	0	(0)	0	(0)
Late										
Esophageal perforation	0	(0)	3	(6)	0	(0)	0	(0)	0	(0)
Esophageal hemorrhage	0	(0)	0	(0)	0	(0)	0	(0)	1	(2)

### Survival

Median follow-up time for all patients was 16.9 months (range 2.6–36.1 months). At the time of this analysis, 22 patients were alive and free of recurrence and 6 was alive with disease. Of the 23 deaths, 20 were related to disease recurrence/progression, 1 for upper gastrointestinal hemorrhage, 1 for comorbid condition (cerebral hemorrhage), and 1 for unknown reasons.

**Fig. 3:**
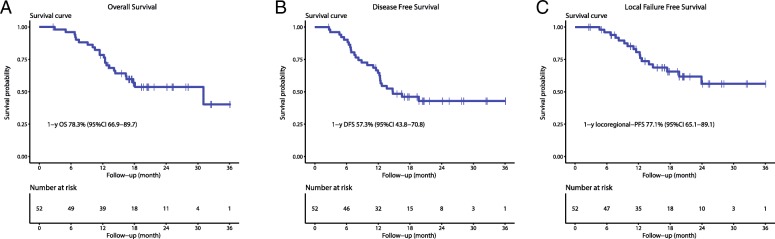
Kaplan-Meier curves. **a** Overall Survival (OS) **b** Disease-Free Survival (DFS) **c** Local Relapse-Free Survival (LRFS)

OS, DFS and local failure-free survival over time are shown in. Figure 3. The median OS time for the entire group was 31 months (95% confidence interval [CI] 10.7–51.4 months) with a 1-year OS rate of 76.9%. The median DFS time for the entire group was 14.7 months (95% confidence interval [CI] 7.5–22.0months) with a 1-year DFS rate of 63.3%. For all patients, the median time to local relapse has not been reached and the 1-year local relapse-free survival was 78.8%.

## Discussion

Compared with other Phase I/II studies using SIB technique [13–16], the dose of our PGTV is lower. The following factors may contribute to lower MTD: Firstly, nearly half (42%) patients treated in our trial were stage IV patients with supraclavicular or abdominal lymph node metastasis (AJCC 6th), while most patients in other clinical trials were stage II or III patients. Secondly, as mentioned above, we adopted ENI and included more prophylactic lymph node drainage areas (supraclavicular, mediastinal and paracardial lymph nodes, etc.) in the CTVs, which differs from other institutions. Besides, the chemotherapy regimen adopted in this trial (paclitaxel and nedaplatin) was different from the most commonly-used regimen in other trials (the combination of platin and flurouracil).

Among these three factors, both advanced disease and ENI could contribute to a larger irradiation field and worse treatment-tolerance: the median volume of PTV in our trial is 630.91cm^3^ (range: 325.14–854.99cm^3^), which is more than three times that of Chen’s trial [15] (median PTV: 199.5cm^3^, range: 97.5–750.7cm^3^). The benefit of ENI for EC patients remains controversial and there is also a lack of consensus on the design of an optimal radiation field. In accordance with the concept of 3-field lymph node dissections in curative surgery, ENI has been adopted for a long time and proven to be an effective way for preventing regional nodal failure [19]. Although the benefit of ENI is now being questioned after publication of some recent retrospective and prospective results [20, 21], we believe that this conclusion still needs confirmation from further large-scale randomized phase III studies. Especially for patients with an advanced disease stage or extensive lymph node metastasis, the utilization of ENI needs further exploration.

However, although the MTD obtained in our phase I/II trial is lower, our survival results (1-y OS reached 76.9%, 1-y local relapse-free survival 78.8%) are comparable to that of other concurrent studies. At the same time, we also achieved a satisfactory compliance rate - 80% patients completed radiotherapy as planned in phase II. Especially considering the relatively advanced disease stage of our patients, it can be said that this dose mode (95% PGTV/PTV 59.92Gy/50.40Gy/28f) concurrent with double-drug chemotherapy is a feasible, safe and effective treatment regimen for patients with advanced EC. Further multi-center Phase III researches have already been started to investigate the application of regimen in a larger-scale population (ClinicalTrials.gov, NCT02979691, NCT03308552, NCT03328234).

## Conclusion

Our study found that for patients with advanced esophageal disease or for centers adopting ENI, it is safe and feasible to give boost area a dose of 59.92Gy/2.14Gy/28f when implementing SIB concurrent with dual-drug chemotherapy. This treatment regimen leads to a satisfactory completion rate and survival results with acceptable toxicity profile.

## Additional file

Additional file 1: **Table S1.** Dose constraints to OARs. (DOCX 13 kb)

## Abbreviations

2DRT: Two-dimensional radiotherapy
3DRT: Three-dimensional radiotherapy
AJCC: American Joint Committee on Cancer
ALP: Alkaline phosphatase
CCRT: Concurrent chemoradiotherapy
CT: Computed tomography
CTCAE: Common terminology criteria for adverse events
CTV: Clinical target volume
DFS: Disease-free survival
EC: Esophageal cancer
ENI: Elective nodal irradiation
EUS: Endoscopic ultrasonography
GTV-N: Metastatic regional nodes
GTV-T: Gross tumor volume
IFRT: Involved field radiotherapy
IMRT: Intensity modulated radiotherapy
KPS: Karnofsky performance score
MDT: Multi-disciplinary team
OAR: Organs at risk
OS: Overall survival
PET: Positron emission tomography
PGTV: Planning gross tumor volume
SCC: Squamous cell carcinoma
SIB: Simultaneous integrated boost
TNM: Tumor-node-metastasis
