# Supplementary material for: A phase I/II radiation dose escalation trial using simultaneous integrated boost technique with elective nodal irradiation and concurrent chemotherapy for unresectable esophageal Cancer
Source: Radiat Oncol. 2019 Mar 15;14:48. doi: 10.1186/s13014-019-1249-5 (PMC6420772; doi:10.1186/s13014-019-1249-5)
Supplement: Supplementary file 1 — Table S1. Dose constraints to OARs. (DOCX 13 kb) [file 13014_2019_1249_MOESM1_ESM.docx]

Additional file 1: Table S1 Dose constraints to OARs

| Organs at Risk (OARs) | Dose constraints |
| --- | --- |
| Lungs | V20＜28%, Dmean＜15Gy |
| Heart | V30＜40%, V40＜30% |
| Cord PRV | Dmax＜45Gy |
| Stomach | V40＜40%, Dmax＜55-60Gy |
| Small intestine | V40＜40%, Dmax＜55Gy |
| Kidneys | V20＜30% |
| Liver | V30＜30% |
